# Effects of Promoter on Structural and Surface Properties of Zirconium Oxide-Based Catalyst Materials

**DOI:** 10.3390/molecules25112619

**Published:** 2020-06-04

**Authors:** Ekaterina S. Borovinskaya, Steffen Oswald, Wladimir Reschetilowski

**Affiliations:** 1 Institute of Industrial Chemistry, Technical University of Dresden, 01062 Dresden, Germany; wladimir.reschetilowski@tu-dresden.de; 2 Saint-Petersburg State Institute of Technology, Technical University, St.-Petersburg 190013, Russia; 3 Leibniz Institute for Solid State and Materials Research, 01062 Dresden, Germany; s.oswald@ifw-dresden.de

**Keywords:** zirconium oxide, copper species, ZnO promoter, NiO promoter, one-pot synthesis, catalyst surface

## Abstract

Ternary mixed oxide systems CuO/ZnO/ZrO_2_ and CuO/NiO/ZrO_2_ were synthesized by one-pot synthesis for a better understanding of the synthesis-property relationships of zirconium oxide-based catalyst materials. The prepared mixed oxide samples were analysed by a broad range of characterisation methods (XRD, N_2_-physisorption, Temperature-Programmed Ammonia Desorption (TPAD), and XPS) to examine the structural and surface properties, as well as to identify the location of the potential catalytically active sites. By XPS analysis, it could be shown that a progressive enrichment of the surface composition with copper takes place by changing from ZnO to NiO as a promoter. Thus, by addition of the second component, not only electronic but also the geometric properties of active sites, i.e., copper species distribution within the catalyst surface, can be affected in a desired way.

## 1. Introduction

Today, the development of new resource-efficient processes is indispensable to provide energy in the future. In this context it is of great importance to develop novel, heterogeneous catalysts for production of liquid fuels derived from greenhouse gas carbon dioxide. Olah et al. [1,2,3], Reschetilowski [4], and other authors [5,6] described methanol production from CO_2_ as advantageous using non-fossil energy sources, avoiding the CO_2_ sequestration and effectively recycling of it to reduce the greenhouse effect. Technical, social, and economic scenario increasingly favours methanol production via conversion of carbon dioxide from various sources using electrolytic hydrogen.

Several catalysts for the hydrogenation of the carbon dioxide have been reported in literature in recent decades [7,8,9,10,11]. CuO/ZnO/Al_2_O_3_ is the conventional catalyst for methanol synthesis from syngas. The action of Cu/Zn-containing catalyst systems is primarily based on the ability of metallic copper to adsorb CO_2_ and hydrogen even at low temperatures. Copper on the ZnO surface is present as small Cu^0^ and Cu^+^ species in the form of active state Cu/CuO pairs, providing an increase of copper surface and its continuous regeneration or maintenance under process conditions. Furthermore, ZnO acts as a promoter by providing the H^δ+^- and H^δ−^-ions required for the catalytic process [12].

Previous mechanistic investigations using in situ IR spectroscopy, DRIFTS (Diffuse Reflectance Infrared Fourier Transform Spectroscopy), and thermal desorption of CO_2_ and of CO_2_/H_2_ gas mixture on the suitable catalysts indicated that formate species are formed on the copper surface, which can be considered as an intermediate of the methanol synthesis [13,14]. The hydrogenolysis of these species with active hydrogen is a rate-limiting step in the methanol formation. According to this reaction mechanism, copper undergoes oxidation/reoxidation cycles and must therefore be easily accessible to all the reactants. However, due to product water, a rapid Cu sintering and catalyst deactivation takes place [15].

Hence, suitable promoters and supports are essential as Cu-based catalysts for methanol synthesis. They play an important role in modulating of interactions between active components. Promoters can, for example, significantly influence the electronic, geometric or acid/base surface properties and thereby the activity and selectivity of the catalyst. In addition, a suitable support is capable to tune or influence the interactions between the active component and the promoter. For example, instead of ZnO promoter as a hydrogen activator NiO or partially reduced Ni in combination with copper can be used to catalyse hydrogenation reactions very effectively. Indeed, catalysts consisting of Cu and Ni were reported to improve catalytic activity in hydrogenation reactions. More important, the nanocomposite nature of catalysts could also help to prevent Cu sintering, even after long reaction time, without loss of activity. It was found out that, in the catalyst Ni(20)Cu(60)-SiO_2_, the well-dispersed metallic Cu species provided the most active phase in the hydrogenation reaction [16].

On the other hand, a suitable support is able to tune or to influence the interactions between the active component and the promoter. For example, the suitability of zirconium (IV) oxide as a catalyst support for methanol synthesis was investigated in literature [17]. Authors reported that zirconium (IV) oxide is a promising catalyst support for methanol synthesis because of its high stability under reducing or oxidizing atmospheres. The presence of ZrO_2_ can also enhance the desired surface properties, as well as copper dispersion, which strongly affects the CO_2_ activation and methanol selectivity. As a result, an increased catalytic activity in comparison to other support materials, e.g., Al_2_O_3_ or SiO_2_, can be observed [18,19,20,21]. Due to higher concentration of oxygen defect sites ZrO_2_-supported catalysts are characterized by a much higher catalytic activity. These materials create novel synergies on the catalyst surface, which are not comprehensively understood due to complexity of the ternary system [22,23,24].

In this context, ZrO_2_-based supported mixed oxide systems CuO/ZnO and CuO/NiO by one-pot synthesis, using ZnO or NiO as a structural and electronical promoter for catalytic active copper species, were synthesised. In such multi-component systems, the promoter influences the catalytic properties of active metal species, providing changes in its dispersion and electronic properties, or may be directly involved in the reaction. The aim of this work was, therefore, to investigate the effects of various promoters on structural and surface chemical properties of the potential catalytically active metal species in ZrO_2_-based catalytic materials.

## 2. Results and Discussion

### 2.1. X-ray Diffraction (XRD)

**Figure 1:**
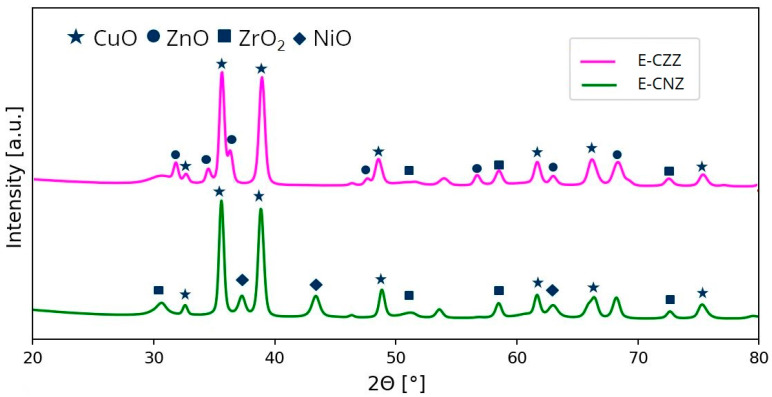
X-ray diffraction pattern of the calcined samples.

XRD investigations revealed that the prepared samples are mixed oxide systems, with typical reflexes in the X-ray diffraction pattern clearly visible for the main components CuO, ZnO, or NiO and ZrO_2_ (Figure 1). The most notable diffraction peaks in XRD patterns of the samples occurred around 2θ = 32.5°, 35.5°, 38.6°, 48.8°, 61.6°, and 66.3°. According to literature, these peaks can be provided by the monoclinic structure of CuO (JCPDS card No. 48-1548) [25].

Likewise, both samples showed in XRD pattern reflexes at 2θ = 30.3°, 50.7°, and 59.8°, which indicate the presence ZrO_2_ tetragonal phase (JCPDS card No. 17-923). No other ZrO_2_ phases were detected, showing that the sample has the single-phase tetragonal structure. For the sample, E-CZZ reflexes were detected at 2θ = 31.8°, 34.3°, 36.2, 47.54°, 56.6°, 62.8°, and 67.9°, which can be identified as ZnO with wurtzite phase (JCPDS card No. 43-0002). The X-ray diffraction patterns of the sample E-CNZ indicated reflexes characteristic for the cubic NiO crystallites at about 2θ = 37.1°, 43.1°, and 62.6° (JCPDS card No. 47-1049).

Since no amorphization of the prepared materials or formation of different secondary phases are observed, according to the literature, reliable particle size determination can be assumed using the Scherrer equation from the X-ray diffraction pattern [26,27]. Based on the obtained diffractograms and the Scherrer equation, it is possible to determine the crystal size of the active components (in this case, CuO) [28].

(1) $$D_{i}=\frac{K\cdot \lambda }{\beta \cdot \cos\left(\theta_{0}\right)},$$

where D_i_—the mean crystal size of the active component, K—the Scherrer form factor, λ—the wavelength of X-rays, β—the full width at half maximum of the reflex, and θ_0_—the Bragg angle.

The Scherrer form factor is defined as K = 0.94, according to the literature [29]. The X-ray wavelength is 0.1541 nm. The full width at half maximum was determined by means of analysis software. In order to obtain more accurate results, the CuO reflex at θ = 38.6° was used, since it is strongly pronounced in both diffractograms, not overlapped, and could therefore be properly analyzed. In addition, the crystal size was corrected with regard to the device-specific reflex broadening. The crystal sizes of CuO determined in this way are summarized in Table 1.

The CuO crystal sizes of the prepared mixed oxide systems were in a range from 8.3 to 8.6 nm. It can be seen that the CuO crystal sizes of the NiO-containing samples are only slightly smaller than those of the ZnO-containing samples. Nevertheless, NiO seems to favour the formation of smaller CuO crystal sizes, which is obviously related to the textural or structural characteristics of the resulting solid.

### 2.2. N_2_-Physisorption

**Figure 2:**
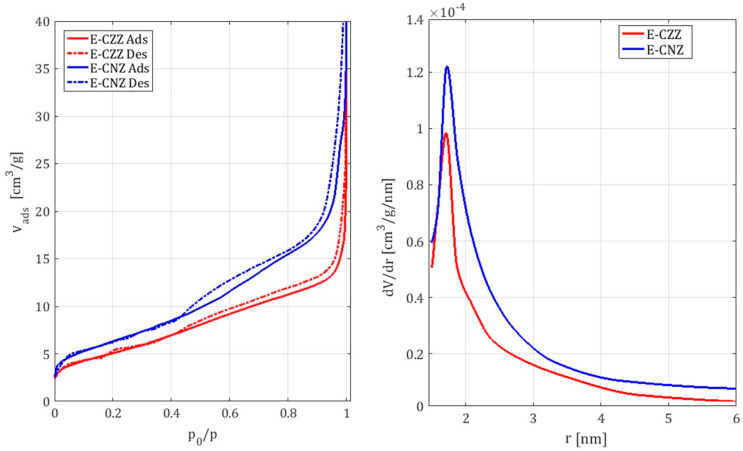
Comparison of the sorption isotherms (**left**) and the pore radius distribution (**right**) of the samples containing ZnO (E-CZZ) and NiO (E-CNZ).

The curves of the nitrogen isotherms for the samples E-CZZ and E-CNZ are presented in Figure 2 (left). The determined BET surface areas (S_BET_) from N_2_-physisorption results are summarized in Table 1.

In general, it can be stated that the investigated ternary mixed oxide systems provide adsorption isotherms of type IV (according to IUPAC). These are characteristic for non-porous or only slightly porous materials. Furthermore, the examined samples show a type H3 hysteresis, which is characteristic for systems with a narrow pore distribution. It is noticeable that the sorption isotherm of the NiO-containing sample is above those of the ZnO-containing sample. This can be explained by the increased specific surface area (Table 1) of the mixed oxide system E-CNZ compared to the sample E-CZZ, which ensures increased adsorption of the adsorptive. Furthermore, the sorption isotherm of the NiO-containing sample shows a sharper increase at low relative pressures. This indicates that it has an increased proportion of micropores.

For a more detailed analysis, the pore radius distribution of the mixed oxide systems according to Barrett, Joyner, and Halenda (BJH method) was determined (Figure 2, right). The well-defined porosity obtained in synthesized materials arises from the slow evaporation of solvents to solid state during the one-pot synthesis. Primary particles in the size of the pore diameter are formed. They block the space behind for the nitrogen molecules during the sorption measurement. Pore blocking might contribute to the low specific surface area of materials. Most of the pores formed are obviously uniform slit pores due to the hysteresis curve H3. They are too small and only the relatively small outer surface is available. The high number of primary particles leads, in turn, to the observed monomodal pore size distribution.

Clear textural differences in term of the specific surfaces, as well as the sorption isotherms and pore radii for obtained ternary mixed oxide systems with different promoters, were observed. The sample with NiO instead of ZnO has a slightly increased specific surface area and an increased proportion of micropores (Table 1).

### 2.3. Temperature-Programmed Ammonia Desorption (TPAD)

Using the TPAD, the total acidity of the solid sample surface can be investigated. This is calculated from the desorbed ammonia depending on test conditions, such as temperature and ammonia volume, used. The corresponding results for the investigated samples are shown in Table 1. Comparing the ZnO- and NiO-containing mixed oxide systems, it is noticeable that the sample E-CNZ has a lower acidity. A possible explanation could be the special affinity of NiO for the tetragonal phase of zirconium (IV) oxide, which has oxygen vacancies and interacts with the NiO more intensively due to the excess of oxygen atoms in the crystal lattice. As a result, Lewis-acidic ZrO_2_ surface sites are blocked and thus are not accessible for ammonia adsorption. On the other hand, this has a positive effect on the morphology and distribution of the corresponding oxide particles, as well as the surface area (Table 1).

Considering the total acidity in combination with the acid site density, it can be noted that the acid site density of the NiO-containing mixed oxide system is only about half as high as that of the ZnO-containing corresponding system (Table 1). This is related to the higher specific surface area and the lower total acidity of this sample.

### 2.4. X-ray Photoelectron Spectroscopy (XPS)

**Figure 3:**
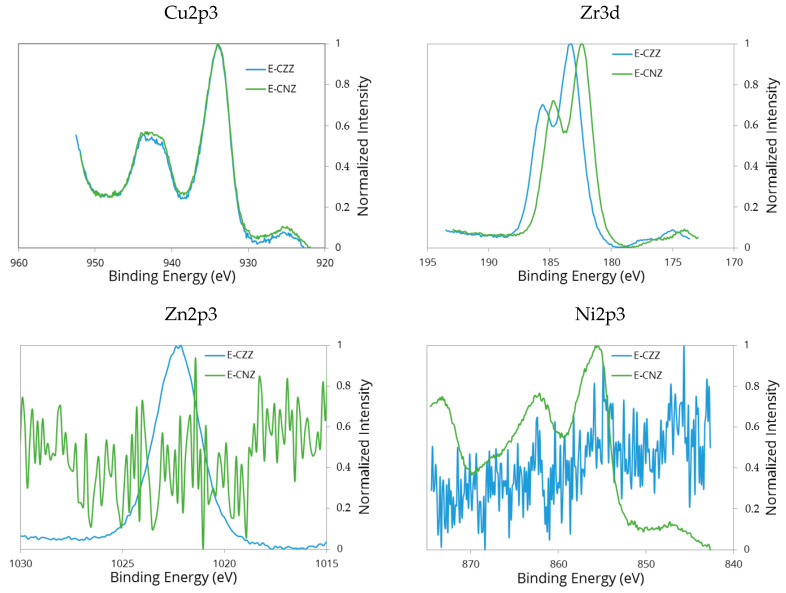
Cu2p_3/2_-, Zn2p_3/2_-, Ni2p_3/2_-, and Zr3d_5/2_ lines in the XPS spectra for E-CZZ (blue) and E-CNZ samples (green).

XPS is an important method for studying the bonding conditions and electronic structure of surface atoms, as well as the chemical composition of solids in the surface region. The XPS spectra of the samples E-CZZ and E-CNZ prepared by the one-pot synthesis are shown in Figure 3.

Position of the Cu2p_3/2_ peak is almost identical in both samples, indicating the same oxidation state of copper in these samples. The value of binding energy of the peaks observed in the Cu2p_3/2_ spectral range are in good agreement with those obtained by other authors [30]. The characteristic (and identical) satellite peak at about 942 eV point to Cu^2+^ can be identified. However, the formation of a higher dispersed CuO phase inside the solids cannot be excluded. This assumption is supported by the fact that a slightly decreased CuO crystal size is observed in the NiO-containing mixed oxide system (Table 1). The agglomeration of it is weakened by an increased NiO-CuO interaction compared to the ZnO-containing sample.

In contrast, the Zr3d_5/2_ line in the NiO-containing sample shows a clear shift towards lower binding energies compared to the ZnO-containing corresponding sample. This might indicate the intensive electronic interaction between NiO and ZrO_2_ already discussed above. Actually, a slight chemical shift of the Ni2p_3/2_ line towards higher binding energies is observed in the XPS spectrum of the NiO-containing sample. The typical Ni^2+^ 2p_3/2_ peak for NiO at 853.7 eV [31] is 3 eV higher than the Ni^2+^ 2p_3/2_ peak observed for unsupported oxide according to a noticeable lowering of the Ni−O bond covalence [32]. This can also mean that some of the Ni^2+^ ions of the crystal lattice are transformed into the Ni^3+^ ions to maintain the electroneutrality. In both Ni^2+^ and Ni^3+^ cases, it is difficult to assign a single binding energy to these chemical states. Nevertheless, it appears that the Ni^3+^ state has a predominance of intensity at higher binding energy in the main signal [33]. This may be related to the valence of Ni cations, as well as particle size of corresponding oxides and the associated surface effects of the catalyst [34].

It is generally assumed that, in the mixed oxide systems under consideration, Cu, Cu/CuO, or Cu/Cu^+^ pairs can act as the most important active component in the hydrogenation of CO_2_ to CH_3_OH, whereby the role of different copper species depending on the used promoters is controversially discussed [35,36,37,38]. Likewise, the nature of the catalytically active Cu species at interface is still in dispute [39,40]. Koeppel et al. [41] found out, based on X-ray diffraction measurements, that active copper species are present predominantly as Cu^0^ over Cu/ZrO_2_. In contrast, Cu^+^ was proposed to be the active component for a Cu/ZnO/SiO_2_ catalyst by performing static low-energy ion scatter experiments [42]. The observed oxidation behavior of Cu species differs clearly from pure metallic Cu. This oxidation behavior and the methanol synthesis activity of the reduced catalyst surface are explained in terms of the formation of Cu^+^/ZnO with oxygen vacancies. However, it was also suggested that Cu metal and low valence of Cu (Cu^n+^ and Cu^+^) may affect the catalytic activity of Cu-based mixed oxide catalysts [43].

Nevertheless, it is generally considered that metallic copper has significant positive influence on the catalytic activity with respect to methanol synthesis despite different environments as an active site. For this reason, after treatment of the samples in a reducing atmosphere, which typically occurs in the CO_2_ hydrogenation, XPS investigations were carried out. The main aim is to consider and to evaluate the influence of the promoting Zn or Ni species on the composition and electronic addition of the active component. This enables the identification of different species on the surface of the ternary mixed oxide systems after reduction and investigation of their interaction with the support in more detail. It can be assumed that the individual components are reducible to varying degrees, and their reducibility is largely determined by particle morphology and distribution, as well as by metal/metal oxide/support interaction. For this purpose, the samples were reduced ex situ under standard conditions. To remove the moisture, the samples were first heated up to 120 °C in dried nitrogen stream (0.2 L/min) in the sample chamber and left at this temperature for one hour. The samples were then treated by a reducing gas mixture H_2_/N_2_ = 1:2.5 at raising temperature up to 220 °C. After completion of a two-hour reduction, the hydrogen was stopped, and the sample chamber was cooled to room temperature. In order to ensure the neutral reaction environment, the sample chamber was flushed out with nitrogen for 30 min and tightly closed. Subsequently, the sample was transferred to the analyzer chamber for the reference- or XPS-measurements.

**Figure 4:**
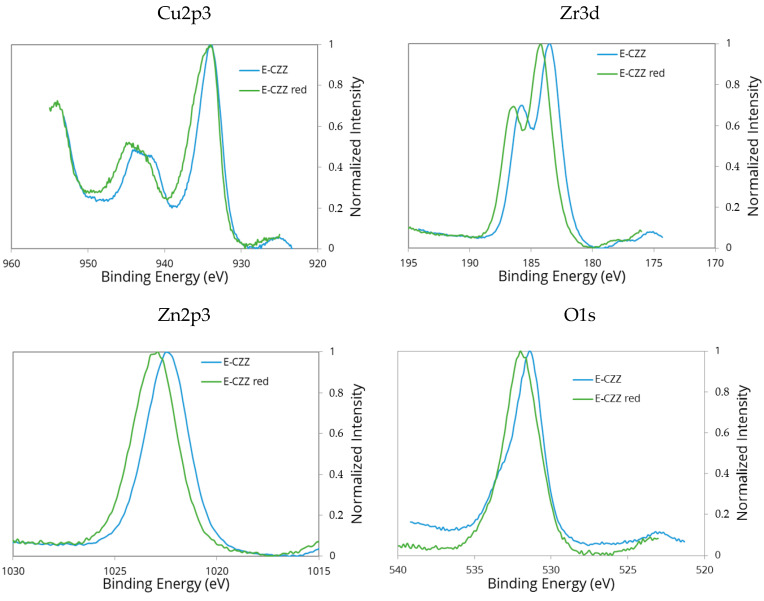
Cu2p_3/2_-, Zn2p_3/2_-, Zr3d_5/2_-, and O1s-lines in the XPS spectra of the calcined (blue) and reduced E-CCZ sample (green).

Binding energies of the main elements of the calcined and reduced mixed oxide system CuO/ZnO/ZrO_2_ are presented in the XPS-spectra in Figure 4. The shift of the Zn2p_3/2_ line to higher binding energies observed in the reduced sample indicates an increased interaction between Zn^2+^ ions and the support surface. This interaction can be related to the presence of defect sites in ZrO_2_ already mentioned above. The number of these sites seems to increase after the reductive treatment of the samples, which is confirmed by the shift to higher binding energies in the O1s spectrum. Furthermore, a shift of the binding energy to higher values in the Zr3d_5/2_ spectrum is consistent with the assumption that there is a strong interaction between the Zn species and the surface sites of the support.

In contrast, almost none or only slight changes are observed in the Cu2p_3/2_ spectrum, so that formation and interaction between Cu^0^ or Cu^n+^ species and the support seem unlikely. This is because, neither with Mg measurements (due to superposition with Zn2p_3/2_, Zr3d_5/2_ and Ni2p_3/2_ peaks) nor with Al source, no significant changes in the Auger peaks and, consequently, no clear valence differences of copper could be observed. The presence of an important satellite peak indicates, without ambiguity, that most of the copper are Cu^2+^ species. The shoulder could be, however, interpreted as Cu2p_3/2_ spectral peak with included partly low-valence copper.

**Figure 5:**
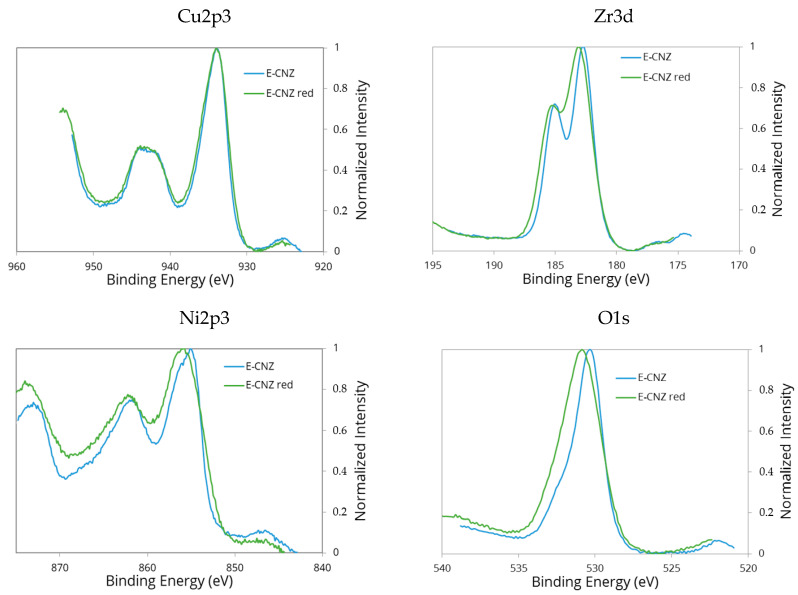
Cu2p_3/2_-, Ni2p_3/2_-, Zr3d_5/2_-, and O1s-lines in the XPS spectra of the calcined (blue) and reduced E-CNZ sample (green).

In the calcined and reduced mixed oxide system CuO/NiO/ZrO_2_ (Figure 5), a slight shift of the Ni2p_3/2_ line to higher binding energies can be observed after reductive treatment. Apparently, the reduction of the samples in this case also leads to an increase of defect sites in the support surface. However, it occurs to a much lesser extent compared to the ZnO-containing mixed oxide system.

The atomic concentration of the elements in the surface layer shows a slight increase in the Cu concentration for the Ni-containing mixed oxide system (Table 2). This can be explained by the fact that, due to an interaction between Ni^n+^ species and support, the outside support surface is covered by CuO at a higher degree. During reduction, this leads to an unequal distribution of Cu^n+^ species in the support and to a slight increase of the Cu concentration in the outer support layers. This is indicated by the resulting decrease in the concentration of O^2-^-anions in the NiO-containing mixed oxide system compared to the ZnO-containing sample.

The peak position and the satellite structure associated with the Cu2p_3/2_ band clearly showed the presence of Cu^2+^ ions, which obviously dissolved in the zirconium oxide lattice and formed highly covalent bindings with oxygen anions. Since the detected copper binding energies for reduced E-CNZ materials are deeper compared to E-CZZ, the copper oxide is located next to the oxygen vacancies on the external surface area of the zirconium oxide support, as suggested above. Thus, the support ZrO_2_ contributes to the stabilization of the CuO phase and decreases the metallic surface but generates local electron-deficient metal particles at the same time, which are apparently necessary for the high methanol selectivity [39,40].

At the phase interface between the copper or low-valence copper and the electronic promoter ZnO or NiO, either a higher activity (due to the accumulation of copper in the outer surface layer) or a lower activity (due to the simultaneous depletion of Ni) can be expected in Ni-containing mixed oxide systems during the CO_2_ hydrogenation. Thus, a balance between these two influencing factors should be considered while developing an effective zirconium oxide-based catalyst system for CO_2_ hydrogenation using NiO as promoter. It can be concluded that the use of NiO instead of ZnO encourage the formation of the better dispersion of Cu species in the outer surface of the solid and thus should promote its catalytic effect. At the same time, the associated reduction of the surface acidic sites density prevents the aggregation of the Cu species, loss and/or changes in its oxidation state, making such materials in this regard comparable with the commercial catalysts for CO_2_ hydrogenation.

**Figure 6:**
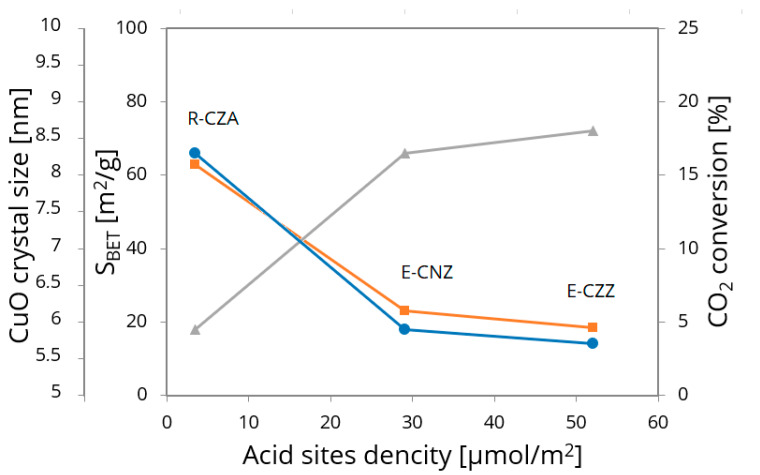
Relationship between BET surface area and acid sites density (orange), as well as CuO crystal size (grey), of prepared mixed oxide systems and their catalytic performance (blue) in comparison with reference catalyst.

Comparison of the prepared mixed oxide systems with a reference catalyst of the same element composition (CuO/ZnO/Al_2_O_3_ (R-CZA), provided by Clariant, Sulzbach (Taunus), Germany) can be gradated with regard to the determined CuO crystallite size: R-CZA (5.9 nm) < E-CNZ (8.3 nm) < E-CZZ (8.6 nm). The density of the surface acidic sites, detected by TPD-NH_3_, changes in the same way: R-CZA (4.20 μmol·NH_3_/m²) < E-CNZ (29.05 μmol·NH_3_/m²) < E-CZZ (52.05 μmol·NH_3_/m²). This agrees with literature results for the reference catalyst, which provides good catalytic activity in the conversion of carbon dioxide and can be characterized not only by higher BET surface area but also by low acid sites density [44,45]. Preliminary catalytic investigations with CO_2_/H_2_ gas mixture (in a ratio of H_2_/CO_2_ = 3) in a plug flow reactor at 220 °C and 1 MPa bar lead to following gradation of catalytic activity: R-CZA > E-CNZ > E-CZZ (Figure 6). Performance of catalytic materials synthesized by the proposed one-pot technique is not yet as good as with the reference catalyst system. Under test conditions, the reference catalyst provided CO_2_ conversion of 16.5%, and the NiO-containing and ZnO-containing ZrO_2_-based system achieved conversion of 4.3 and 3.4%, respectively (Figure 6). Hence, decreasing the acid sites density of the prepared ZrO_2_-based mixed oxide systems by increasing of the surface area could be an interesting tool to generate the required catalyst performance. Further research is needed to elucidate the role of the specific acid sites in the forming of the fine dispersion of Cu species in the outer support surface and in the tuning the electronic properties of the Cu species by combination with suitable promoter [46].

## 3. Materials and Methods 

### 3.1. Materials

In the one-pot synthesis, the nitrates Cu(NO_3_)_2_·xH_2_O (Puratronic^®^, 99.999%, metals basis), Zn(NO_3_)_2_·6H_2_O (Puratronic^®^, 99.998%, metals basis) or Ni(NO_3_)_2_·6H_2_O (Puratronic^®^, 99.998%, metals basis) and ZrO_2_(NO_3_)_2_·xH_2_O (Puratronic^®^, 99.994%, metals basis) were weighted and dissolved in water. The weights of the nitrates used were based on a 10 g catalyst sample of the same composition 60/30/10 wt.-% for CuO/ZnO/ZrO_2_ and CuO/NiO/ZrO_2_, respectively. The resulting solution was evaporated (100 °C for 5 h), and the residue was dried and then calcined.

The calcination of the synthesized samples was carried out in an oven of the *Horststatt* company, on a frit, which was put in a glass flask was by means of ground clamps. An equivalent mass of sample material was used for each calcination performed. The calculation was carried out according to the following temperature program: heating rate 1.2 K/min → 120 °C, holding time 1 h → 540 °C, holding time 12 h.

The prepared catalyst samples were named as follows: Sample name used was based on the preparation method (E—one-pot synthesis) and the components used (CuO, ZnO, or NiO and ZrO_2_) as C, Z, or N and Z. The designation E-CZZ means, that this sample was synthesized by the one-pot synthesis (E) and contains 60 wt.-% CuO (C), 30 wt.-% ZnO (Z) and 10 wt.-% ZrO_2_ (Z). On the other hand, the name E-CNZ means, that this sample was synthesized in the same way (E) and contains also 60 wt.-% CuO (C), 30 wt.-% NiO (N) and 10 wt.-% ZrO_2_ (Z).

### 3.2. Methods

XRD patterns of the prepared samples were achieved with the SuperNova single crystal diffraction instrument (Rigaku Oxford Diffraction, Kent, U.K.) using CuK_α_ radiation. Nitrogen adsorption-desorption isotherms were measured at 77 K on a Sorptomatic 1990 (Carlo Erba Instruments, Egelsbach, Germany). Acidic properties were analyzed by temperature-programmed desorption of ammonia. After the sample pretreatment, including calcination, ammonia adsorption and removing of physisorbed ammonia, the surface properties were analyzed by in situ IR-spectroscopy (Nicolet Impact 400, Waltham, Massachusetts, U.S.) and mass spectrometry in the range of 373 to 823 K. XPS measurements were carried out at a PHI 5600 CI spectrometer (Physical Electronics, Feldkirchen, Germany), which is equipped with a hemispherical energy analyzer. Pass energy used was 29 eV, the measuring area is around 800 µm in diameter. Due to strong charging effects measurements with monochromatic X-rays war were not possible, non-monochromatic MgK_α_ source (400 W) was used. Residual charging shifts, which are not identical in each sample, have to be corrected using internal reference peaks. The mostly used C1s peak from contamination was not reliable because of the low carbon content, and the Cu2p_3/2_ peak (available in all samples) at 934 eV was used.

## 4. Conclusions

The potential catalytically active ternary mixed oxide systems on the zirconium oxide support were synthesized by the one-pot synthesis. All necessary catalyst precursors were simultaneously converted by evaporation from solutions into the solid state. The synthesized samples were characterized by physical-chemical measuring methods. By means of XRD, it could be confirmed that all systems correspond to desired ternary mixed oxides with high purity. The adsorption capacity of the examined samples and their texture, i.e., surface area and pore size, were determined by nitrogen adsorption. In this case, the NiO-containing system showed a slightly higher surface area compared to ZnO-containing sample with a similar pore size. The larger surface area of the sample with the promoter NiO can support the formation of slightly smaller CuO crystals. On the other hand, the TPAD measurements showed that the NiO-containing sample is characterized by a lower total acidity. Consequently, also due to the higher surface area, the density of acidic sites in this sample is lower than in the corresponding ZnO-containing sample. XPS measurements confirmed that there is an increased interaction with both the ZrO_2_ support and the active component copper in the NiO-containing sample. Ni species accumulate especially in an inner solid layer, while Cu species accumulate in the outer solid surface. In agreement with observations reported in literature, it can be concluded that the ternary mixed oxide systems based on ZrO_2_ with high surface area, low density of acidic sites, and distribution of the active component in the outer catalyst layer are advantageous for high catalytic activity in the CO_2_ hydrogenation.

## Figures and Tables

**Table 1:** Summary of the data obtained from XRD, N_2_-physisorption, and Temperature-Programmed Ammonia Desorption (TPAD).

Sample	CuO Crystal Size [nm]	S_BET_ [m²/g]	Total Acidity [mmol·NH_3_/g]	Acid Site Density [μmol/m²]
E-CZZ	8.6	18.5	0.963	52.05
E-CNZ	8.3	23.1	0.671	29.05

**Table 2:** Comparison of the atomic concentration in the outer support layers for the calcined and reduced samples E-CZZ and E-CNZ.

Sample	O1s	Ni2p3	Cu2p3	Zn2p3	Zr3d
E-CZZ	56.9	0.00	17.3	11.7	14.1
E-CZZ_red_	55.6	0.00	17.4	12.3	14.7
E-CNZ	52.7	17.8	19.6	0.0	9.9
E-CNZ_red_	52.5	15.4	19.4	0.0	12.7

## References

[1] Olah G.A., Goeppert A., Prakash G.K.S. (2009). Beyond Oil and Gas: The Methanol Economy.

[2] Olah G.A., Prakash G.K.S., Goeppert A. (2011). Anthropogenic chemical carbon cycle for a sustainable future. J. Am. Chem. Soc..

[3] Olah G.A., Goeppert A., Prakash G.K.S. (2009). Chemical recycling of carbon dioxide to methanol and dimethyl ether: From greenhouse gas to renewable, environmentally carbon neutral fuels and synthetic hydrocarbons. J. Org. Chem..

[4] Reschetilowski W. (2013). Alternative resources for the methanol economy. Russ. Chem. Rev..

[5] Aresta M., Dibenedetto A., Angelini A. (2013). The changing paradigm in CO_2_ utilization. J. CO_2_ Util..

[6] Song C. (2006). Global challenges and strategies for control, conversion and utilization of CO_2_ for sustainable development involving energy, catalysis, adsorption and chemical processing. Catal. Today.

[7] Jandhav S.G., Vaidya P.D., Bhanage B.M., Joshi J.B. (2014). Catalytic carbon dioxide hydrogenation to methanol: A review of recent studies. Chem. Eng. Res. Des..

[8] Su C., Li J., He D., Cheng Z., Zhu Q. (2000). Synthesis of isobutene from synthesis gas over nanosize zirconia catalysts. Appl. Catal. A Gen..

[9] Liu J., Shi J., He D., Zhang Q., Wu X., Liang Y., Zhu Q. (2001). Surface active structure of ultra-fine Cu/ZrO_2_ catalysts used for the CO_2_ + H_2_ to methanol reaction. Appl. Catal. A Gen..

[10] Denise B., Sneeden R.P.A. (1986). Oxide-supported copper catalysts prepared from copper formate: Differences in behaviour in methanol synthesis from CO/H_2_ and CO_2_/H_2_ mixtures. Appl. Catal..

[11] Amenomiya Y. (1987). Methanol synthesis from CO_2_ + H_2_ II. Copper-based binary and ternary catalysts. Appl. Catal..

[12] Chinchen G.C., Waugh K.C., Whan D.A. (1986). The activity and state of the copper surface in methanol synthesis catalysts. Appl. Catal..

[13] Borovinskaya E., Trebbin S., Alscher F., Breitkopf C. (2019). Synthesis, modification and characterization of CuO/ZnO/ZrO_2_ mixed oxide catalysts for CO_2_/H_2_ conversion. Catalysts.

[14] Grabow L.C., Mavrikakis M. (2011). Mechanism of methanol synthesis on Cu through CO_2_ and CO hydrogenation. ACS Catal..

[15] Natesakhawat S., Lekse J.W., Baltrus J.P., Ohodnicki P.R., Howard B.H., Deng X., Matranga C. (2012). Active sites and structure-activity relationships of copper-based catalysts for carbon dioxide hydrogenation to methanol. ACS Catal..

[16] Upare P.P., Jeong M.-G., Hwang Y.K., Kim D.H., Kim Y.D., Hwang D.W., Lee U.-H., Chang J.-S. (2015). Nickel-promoted copper-silica nanocomposite catalysts for hydrogenation of levulinic acid to lactones using formic acid as a hydrogen feeder. Appl. Catal. A Gen..

[17] Wang Y., Kattel S., Gao W., Li K., Liu P., Chen J.G., Wang H. (2019). Exploring the ternary interactions in Cu–ZnO–ZrO_2_ catalysts for efficient CO_2_ hydrogenation to methanol. Nat. Commun..

[18] Arena F., Barbera K., Italiano G., Bonura G., Spadaro L., Frusteri F. (2007). Synthesis, characterization and activity pattern of Cu–ZnO/ZrO_2_ catalysts in the hydrogenation of carbon dioxide to methanol. J. Catal..

[19] Lachowska M., Skrzypek J. (2004). Methanol synthesis from carbon dioxide and hydrogen over Mn-promoted copper/zinc/zirconia catalysts. React. Kinet. Catal. Lett..

[20] Arena F., Italiano G., Barbera K., Bordiga S., Bonura G., Spadaro L., Frusteri F. (2008). Solid-state interactions, adsorption sites and functionality of Cu–ZnO/ZrO_2_ catalysts in the CO_2_ hydrogenation to CH_3_OH. Appl. Catal. A Gen..

[21] Wang Y.H., Gao W.G., Wang H., Zheng Y.E., Na W., Li K.Z. (2017). Structure-activity relationships of Cu–ZrO_2_ catalysts for CO_2_ hydrogenation to methanol: Interaction effects and reaction mechanism. RSC Adv..

[22] Ren J., Qin X., Yang J.-Z., Qin Z.-F., Guo H.-L., Lin J.-Y., Li Z. (2015). Methanation of carbon dioxide over Ni–M/ZrO_2_ (M=Fe, Co, Cu) catalysts: Effect of addition of a second metal. Fuel Process. Technol..

[23] Pérez-Hernández R., Mondragón Galicia G., Mendoza Anaya D., Palacios J., Angeles-Chavez C., Arenas-Alatorre J. (2008). Synthesis and characterization of bimetallic Cu–Ni/ZrO_2_ nanocatalysts: H_2_ production by oxidative steam reforming of methanol. Int. J. Hydrog. Energy.

[24] Wolfbeisser A., Klötzer B., Mayr L., Rameshan R., Zemlyanov D., Bernardi J., Föttinger K., Rupprechter G. (2015). Surface modification processes during methane decomposition on Cu-promoted Ni–ZrO_2_ catalysts. Catal. Sci. Technol..

[25] International Centre for Diffraction Data (ICDD) (2000) Joint Committee on Powder Diffraction Standards, Diffraction Data File No. 05-0661, 12–23. https://www.icdd.com/.

[26] Langford J.I., Wilson A.J.C. (1978). Scherrer after sixty years: A survey and some new results in the determination of crystallite size. J. Appl. Crystallogr..

[27] Pecharsky V.K., Zavalij P.Y. (2005). Fundamentals of Powder Diffraction and Structural Characterization of Materials.

[28] Hall B.D., Zanchet D., Ugarte D. (2000). Estimating nanoparticle size from diffraction measurements. J. Appl. Crystallogr..

[29] Speakman S.A. (2014). Estimating Crystallite Size Using XRD.

[30] XPS Interpretation of Copper. https://xpssimplified.com/elements/copper.php.

[31] XPS Interpretation of Nickel. https://xpssimplified.com/elements/nickel.php.

[32] Davidson A., Tempere J.F., Che M., Roulet H., Dufour G. (1996). Spectroscopic studies of Nickel(II) and Nickel(III) species generated upon thermal treatments of nickel/ceria-supported materials. J. Phys. Chem..

[33] Grosvenor A.P., Biesinger M.C., Smart R.S.C., Mc Intyre N.S. (2006). New interpretations of XPS spectra of nickel metal and oxides. Surf. Sci..

[34] Liu X., Zhai Z.-Y., Chen Z., Zhang L.-Z., Zhao X.-F., Si F.-Z., Li J.-H. (2018). Engineering mesoporous NiO with enriched electrophilic Ni^3+^ and O^−^ toward efficient oxygen evolution. Catalysts.

[35] Kanai Y., Watanabe T., Fujitani T., Uchijima T., Nakamura J. (1996). The synergy between Cu and ZnO in methanol synthesis catalysts. Catal. Lett..

[36] Fujitani T., Nakamura J. (2000). The chemical modification seen in the Cu/ZnO methanol synthesis catalysts. Appl. Catal. A Gen..

[37] Frost J.C. (1988). Junction effect interactions in methanol synthesis catalysts. Nature.

[38] Burch R., Chappell R.J. (1988). Support and additive effects in the synthesis of methanol over copper Catalysts. Appl. Catal..

[39] Wang W., Wang S., Ma X., Gong J. (2011). Recent advances in catalytic hydrogenation of carbon dioxide. Chem. Soc. Rev..

[40] Guil-López R., Mota N., Llorente J., Millán E., Pawelec B., Fierro J.L.G., Navarro R.M. (2019). Methanol Synthesis from CO_2_: A Review of the Latest Developments in Heterogeneous Catalysis. Materials.

[41] Koeppel R.A., Baiker A., Wokaun A. (1992). Copper/zirconia catalysts for the synthesis of methanol from carbon dioxide: Influence of preparation variables on structural and catalytic properties of catalysts. Appl. Catal. A Gen..

[42] Jansen W.P.A., Beckers J., Van der Heuvel J.C., Van der Gon A.W.D., Bliek A., Brongersma H.H. (2002). Dynamic Behavior of the Surface Structure of Cu/ZnO/SiO2 Catalysts. J. Catal..

[43] Saito M., Fujitani T., Takeuchi M., Watanabe T. (1996). Development of copper/zinc oxide-based multicomponent catalysts for methanol synthesis from carbon dioxide and hydrogen. Appl. Catal. A Gen..

[44] Borovinskaya E., Alscher F., Breitkopf C., Ernst S., Beller M. (2018). Modified zirconium oxide catalysts for effective conversion of carbon dioxide into useful liquid compounds. Preprints of the DGMK Conference “Challenges for Petrochemicals and Fuels: Integration of Value Chains and Energy Transition”.

[45] Zurbel A., Kraft M., Kavurucu-Schubert S., Bertau M. (2018). Methanol Synthesis by CO_2_ Hydrogenation over Cu/ZnO/Al_2_O_3_ Catalysts under Fluctuating Conditions. Chem. Ing. Tech..

[46] Scotti N., Bossola F., Zaccheria F., Ravasio N. (2020). Copper-Zirconia Catalysts: Powerful Multifunctional Catalytic Tools to Approach Sustainable Processes. Catalysts.

